# Solitary thyroid metastasis from colorectal cancer: Case report

**DOI:** 10.1016/j.ijscr.2021.105804

**Published:** 2021-03-22

**Authors:** Hwansoo Kim

**Affiliations:** Department of Surgery, Kangwon National University Hospital, Kangwon National University Hospital, 156, Baengnyeong-ro, Chuncheon-Si, Gangwon-Do 24289, Republic of Korea

**Keywords:** Thyroid metastasis, Colorectal cancer, Case report

## Abstract

**Introduction and importance:**

Thyroid metastasis of colorectal cancer is rare and the patient with thyroid metastasis has no symptoms early in the disease course. On the other hand, evaluation of thyroid is not generally included in the routine follow-up of colorectal cancer. Therefore, the diagnosis of thyroid metastasis of colorectal cancer may be delayed.

**Presentation of case:**

Chest computed tomography revealed a nodule on the right lobe of the thyroid in 53-year-old woman diagnosed with sigmoid colon adenocarcinoma. This nodule was identified having as increased metabolic activity by ^18^F-fluorodeoxyglucose positron emission tomography-computed tomography and confirmed to be consisted of cystic and solid portions by ultrasonography. Fine needle aspiration cytology of the nodule revealed metastasis of colon cancer. The patient underwent laparoscopic anterior resection and a total thyroidectomy. The size of the thyroid tumor was 1.2 cm with free resection margin.

**Discussion:**

The clinical features of thyroid metastasis from colorectal cancer are not typical. In addition, follow-up examinations of patients with colorectal cancer do not include imaging studies of the neck, so the diagnosis of thyroid metastases is delayed. The management strategies for thyroid metastasis from colorectal cancer include a total thyroidectomy without prophylactic neck lymph node dissection and chemotherapy. However, as the penetration of chemotherapeutic drugs into the thyroid is difficult, the effect on the thyroid is considered insignificant.

**Conclusion:**

Surgical treatment should be considered for treatment of thyroid metastasis.

## Highlights

• Thyroid metastasis of colorectal cancer is rare.
• Attention should be paid to the thyroid of colon cancer patients.
• Treatment of thyroid metastasis of colorectal cancer; total thyroidectomy.

## 1 Introduction

Thyroid metastasis of non-thyroid malignancies is reported in 1.4–3% of all patients who undergo surgery for thyroid neoplasms [1]. Thyroid metastasis of colorectal cancer is also rare and occurs late in the disease course. It was reported by Liévre et al. that 6 of 5862 patients with colorectal cancer between 1993 and 2004 were found to have thyroid metastasis [2]. Patients with thyroid metastases rarely develop symptoms early in the disease course [3]. Additionally, thyroid ultrasonography (US) and computed tomography (CT) are not commonly included in routine follow-up examinations of colorectal cancer. Therefore, diagnosis of thyroid metastasis of colorectal cancer may be delayed. We report a case of thyroid metastasis discovered incidentally during an initial staging work-up of a patient with colon cancer.

The work has been reported in line with the SCARE 2020 criteria [4].

## 2 Case report

**Fig. 1:**
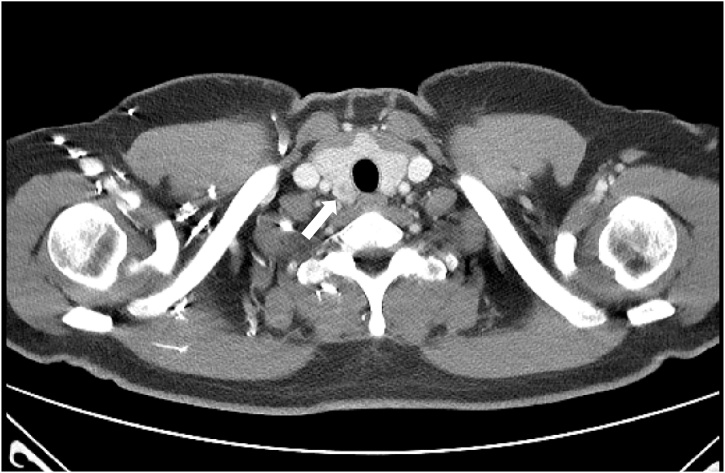
Chest computed tomography scan: a hypodense nodule in the right lobe of thyroid gland (white arrow).

**Fig. 2:**
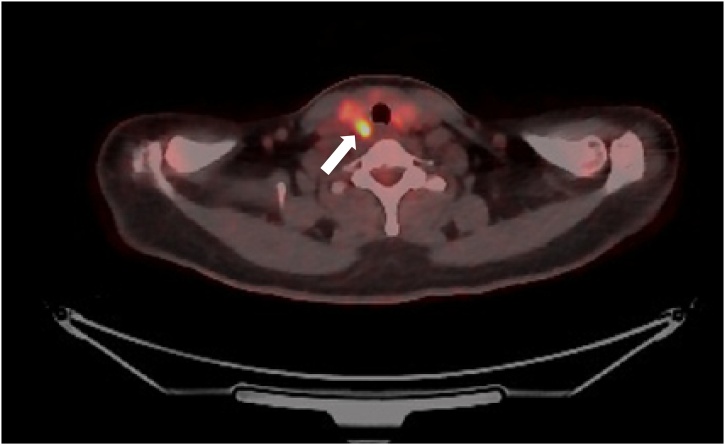
^18^F-fluorodeoxyglucose positron emission tomography-computed tomography (^18^FDG PET-CT): abnormally increased ^18^F-FDG activity in the right lobe of thyroid (white arrow).

**Fig. 3:**
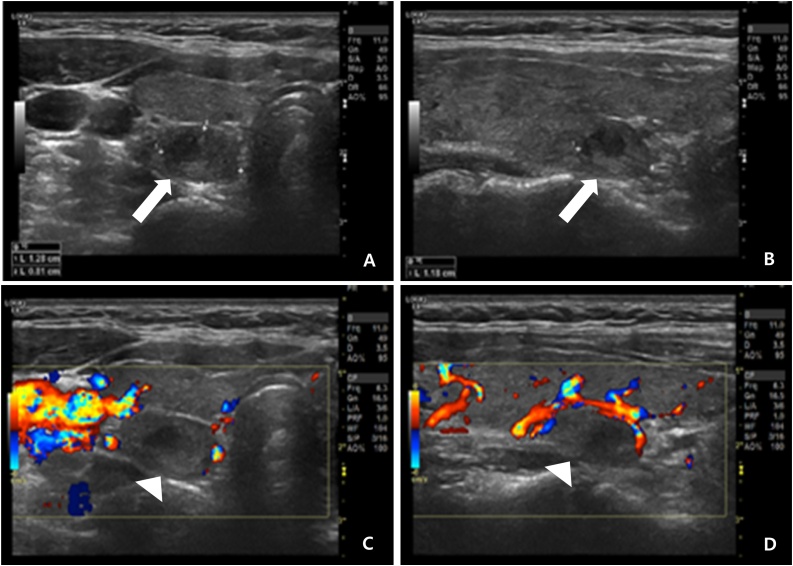
Thyroid ultrasonography: 1.3 × 0.8 × 1.2 cm-sized partially cystic solid nodule (A, B; white arrow) without increasing vascularity (C, D; white arrow head) in the right lobe of the thyroid gland.

In July 2017, a 53-year-old woman who had not past surgical history and any other chronic diseases was diagnosed with sigmoid colon adenocarcinoma. In addition, she denied the presence of family and hereditary diseases. Preoperative examination revealed no metastases in the other solid organs, but chest CT revealed a mass in the right lobe of the thyroid (Fig. 1). This nodule was identified as having increased ^18^F-fluorodeoxyglucose activity in the same site by ^18^F-fluorodeoxyglucose positron emission tomography-computed tomography (^18^FDG PET-CT) (Fig. 2). US revealed that both lobes of the thyroid gland were slightly enlarged and heterogeneously echogenic. The nodule in right lobe of the thyroid was 1.3 × 1.2 × 0.8 cm in size and consisted of cystic and solid portions. Consequently, the nodule displayed characteristics of focal inflammation or benign nodules by US (Fig. 3). Fine needle aspiration cytology of the nodule was used to diagnose a suspicious metastasis from colorectal cancer. At this time, carcinoembryonic antigen was increasing (29.1 ng/mL). The patient underwent laparoscopic anterior resection. The size of the tumor was 4.5 cm, and tumor cells invaded to the pericolic adipose tissue (pT3). Lymphovascular tumor embioli were seen, and neural invasion was also found. Two metastatic lymph nodes were confirmed in 39 lymph nodes (pN1b). The patient also underwent total thyroidectomy without prophylactic neck lymph node dissection. Pathologic examination revealed that the size of the tumor was 1.2 cm, with free resection margins. Adjuvant chemotherapy with modified FOLFOX-6 (mFOLFOX-6) and cetuximab was initiated. There was no evidence of recurrence after 42 months of follow-up.

## 3 Discussion

Thyroid metastasis of a non-thyroidal malignancy is an uncommon clinical presentation. In autopsy series, it is observed in only 1.4–3% of cases, and the lungs are the most common site of primary tumors metastatic to the thyroid [5]. However, in a clinical series, the kidney was the most common primary source of thyroid metastases [6].

The rarity of thyroid metastases could be explained by two hypotheses. First, the thyroid receives an extremely abundant supply of arterial blood, and adhesion and implantation of tumor cells are disturbed by the resulting rapid blood flow. Second, the establishment and development of tumor cells may be prevented by the high oxygen saturation and high iodine content in the thyroid [7,8].

The clinical features of thyroid metastasis from colorectal cancer are not typical. Many patients with thyroid metastasis are asymptomatic (31%) and have normal thyroid function [9]. Therefore, the diagnosis of thyroid metastasis is often delayed. It has been reported by Liévre et al. that half of patients with thyroid metastasis were asymptomatic, and another half had neck swelling or cervical masses [2]. Thyroid dysfunction in patients with thyroid metastases is rare, whereas an increase in tumor markers–primarily carcinoembryonic antigen–is common [10,11]. The timing of diagnosis of thyroid metastases varies [12]. In 2013, Froylich et al. summarized that the primary tumor was located in the rectum (41%), the sigmoid colon (33%), the right colon (19%), and the left colon (11%) in 34 patients with thyroid metastases from colorectal cancer. Froylich et al. also reported that thyroid metastases were diagnosed between 6 months and 8 years after colonic resection [13]. The presence of a thyroid nodule is identified by imaging studies (US, CT, magnetic resonance imaging, and ^18^FDG PET-CT), but fine needle aspiration cytology is needed to confirm whether the nodule is a primary thyroid lesion or a metastatic lesion. In general, follow-up examinations of patients with colorectal cancer do not include imaging studies of the neck, so the diagnosis of thyroid metastases is delayed.

The management strategies for thyroid metastasis from colorectal cancer include surgical treatment and chemotherapy. The extent of metastatic disease in other sites and the medical condition of the patient must be considered before surgical treatment [7,9,14]. Batson reported that the mean survival of patients who underwent thyroidectomy was longer than for those who did not undergo thyroidectomy [15]. However, another study demonstrated that the overall survival in patients treated non-surgically was not significantly different from patients who underwent thyroidectomy alone or thyroidectomy with adjuvant therapy [16]. Surgical treatment prevents the appearance of symptoms such as dyspnea and dysphagia and thus enhances the patient’s quality of life. The extent of surgical resection for thyroid metastasis is not definite. Because thyroid metastases are usually multifocal and cancer cells may remain in the margins after lobectomy alone, a total thyroidectomy is recommended [6,7,9]. Iodine therapy is not mandatory, because the response of thyroid metastases to iodine therapy is negligible [6]. Although Liévre et al. reported that lymph node metastases were confirmed in all patients who underwent thyroidectomy with central and lateral neck node dissection, on the contrary according to other studies, neck lymph node metastasis is not common in patients with thyroid metastasis [2,6]. In generally, prophylactic neck lymph node dissection is not recommended. As chemotherapy for patients with thyroid metastases is aimed at the treatment of colorectal cancer, chemotherapy regimens such as XELOX, mFOLFOX-6, or FOLFIRI are used [1,17,18]. However, as the penetration of chemotherapeutic drugs into the thyroid is difficult, the effect on the thyroid is considered insignificant [13].

## 4 Conclusion

Thyroid metastasis of colorectal cancer is rarely reported. However, as thyroid metastases are usually asymptomatic, and thyroid studies are not routinely performed after colorectal cancer surgery, thyroid metastasis can remain undiagnosed, and if diagnosed, is often advanced. Therefore, attention should be paid to the thyroid of colon cancer patients. There is no definitive treatment for thyroid metastasis. Nevertheless, surgical treatment is given priority because surgical treatment can be expected to prolong the survival period, improve quality of life, and prevent symptoms such as dyspnea and dysphagia.

## Declaration of Competing Interest

The author reports no declarations of interest.

## Sources of funding

The author received no financial support for the preparation of this case report.

## Ethical approval

The ethical committee approval was not required given the article type (case report).

## Consent

Written informed consent was obtained from the patient for publication of this case report and accompanying images.

## Author contribution

HS Kim: data collection, data analysis or interpretation, writing the paper, references, paper writing and revision, figures revision.

## Registration of research studies

Not applicable.

## Guarantor

Hwansoo Kim is the guarantor of the study and accept full responsibility for the work and/or the conduct of the study, had access to the data and controlled the decision to publish.

## Provenance and peer review

Not commissioned, externally peer-reviewed.
